# Evidence for Dopamine Abnormalities Following Acute Methamphetamine Exposure Assessed by Neuromelanin-Sensitive Magnetic Resonance Imaging

**DOI:** 10.3389/fnagi.2022.865825

**Published:** 2022-05-30

**Authors:** Fei Tang, Hui Liu, Xiao Jie Zhang, Hui Hui Zheng, Yong Ming Dai, Li Yun Zheng, Wen Han Yang, Yan Yao Du, Jun Liu

**Affiliations:** ^1^Department of Radiology, Second Xiangya Hospital of Central South University, Changsha, China; ^2^MR Collaboration, Central Research Institute, United Imaging Healthcare, Shanghai, China

**Keywords:** neuromelanin-sensitive magnetic resonance imaging, dopamine, methamphetamine, substantia nigra, neuromelanin, drug addiction

## Abstract

**Background:**

Neuromelanin-sensitive magnetic resonance imaging (NM-MRI) is a newly developed MRI technique that provides a non-invasive way to indirectly measure of dopamine (DA) function. This study aimed to determine NM concentrations in brain regions following acute methamphetamine (MA) administration using NM-MRI and to explore whether NM-MRI can be used as a biomarker of DA function in non-neurodegenerative diseases.

**Methods:**

Baseline NM-MRI, T1-weighted and T2-weighted images were acquired from 27 rats before drug/placebo injection. The control group (*n* = 11) received acute placebo (Normal saline), while the experimental group (*n* = 16) received acute MA. NM-MRI scans were performed 5, 30, 60 and 90 min after injection. Regions of interest (ROIs), including the caudate putamen (CP), nucleus accumbens (NAc), hippocampus (HIP), substantia nigra (SN) and crus cerebri (CC), were manually drawn by an experienced radiologist. NM-MRI signal intensity in five brain regions at different time points (baseline and 5, 30, 60, and 90 min) were analyzed.

**Results:**

In both the control and experimental groups, at each time point (baseline and 5, 30, 60, and 90 min), the SN exhibited significantly higher NM-MRI signal intensity than the other brain regions (*P* < 0.05). In addition, acute MA administration resulted in a continuous upward trend in NM-MRI signal intensity in each brain region over time. However, there was no such trend over time in the control group. The NM-MRI signal intensity of SN in the experimental group was significantly higher at the 60 and 90 min compared with that in the control group (*P* values were 0.042 and 0.042 respectively). Within experimental group, the NM-MRI signal intensity of SN was significantly higher at the 60 and 90 min compared with that before MA administration (*P* values were 0.023 and 0.011 respectively). Increased amplitudes and rates of NM-MRI signal intensity were higher in the SN than in other brain regions after MA administration.

**Conclusion:**

Our results indicated that NM was mainly deposited in the SN, and the conversion of DA to NM was most significant in the SN after acute MA exposure. Increased DA release induced by acute MA exposure may lead to increased accumulation of NM in multiple brain regions that can be revealed by NM-MRI. NM-MRI may serve as a powerful imaging tool that could have diverse research and clinical applications for detecting pathological changes in drug addiction and related non-neurodegenerative diseases.

## Introduction

The dopaminergic system plays a crucial role in a broad spectrum of neuropsychiatric disorders (e.g., Parkinson’s disease (PD), schizophrenia and addiction) (Poulin et al., 2020), and dopaminergic dysfunction has been hypothesized to underpin drug addiction-related behavior (Lin et al., 2016). Drug addiction is a non-degenerative disease, which results in elevated levels of extracellular monoamine neurotransmitters in all brain regions with a magnitude of increase that varies between brain regions (Zhang et al., 2001; Bubenikova-Valesova et al., 2009). Methamphetamine (MA) is one of the most widely abused psychostimulants that may cause addiction (Shi et al., 2000; Saha et al., 2014). Brain dopaminergic pathways are activated by MA exposure, which promotes dopamine (DA) release from presynaptic nerve endings, inhibits DA transporter (DAT) reuptake, results in increased extracellular DA levels (Cruickshank and Dyer, 2009; Kim et al., 2020), and damages the dopaminergic system in all animal species including humans (Moratalla et al., 2017; Granado et al., 2018). Compulsive drug-seeking and drug-taking are important drug abuse-related behaviors that have been linked to alterations in dopaminergic neurotransmission and to impaired inhibitory control (Groman et al., 2012). Regarding DA, MA activates the mesolimbic and nigrostriatal dopaminergic pathways, and the clinical response to the drug in humans includes euphoria, arousal, reduced fatigue, positive mood, and anxiety. MA administration in rats induces behavioral sensitization which is characterized by augmentation of locomotor activity (Ares-Santos et al., 2014). Behavioral sensitization has been postulated to result from enhanced DA release in the mesolimbic and nigrostriatal dopaminergic terminals. DA cell bodies in the midbrain are the primary source of dopaminergic input to the caudate putamen (CP) and nucleus accumbens (NAc), and DA neurons in the ventral tegmental area (VTA) and the substantia nigra (SN) send their axons to the NAc and CP, respectively (Lammel et al., 2014; Thanos et al., 2016). DA is the major neurotransmitter in the neostriatum (Björklund and Dunnett, 2007), and neural activity of DA neurons in the somatodendritic field may influence the synthesis and release of DA in the terminal regions (Poulin et al., 2020; Lu et al., 2021). *In vitro* studies have shown enhanced DA release from NAc and striatum tissue slices from rats sensitized to psychostimulants such as MA (Desai et al., 2010; Granado et al., 2010). *In vivo* measurement of DA function is critical for understanding how this key neuromodulator contributes to addiction and may provide objective markers of DA function in MA addiction. Currently, positron emission tomography (PET) is the main imaging method to measure DA function (Huang et al., 2019). However, PET is limited by the risk of radioactivity exposure and costly specialized infrastructure. The non-invasive measurement of DA function abnormalities by optimal imaging method remains challenging.

Dopamine neurons contain a melanin pigment known as neuromelanin (NM) that is mainly distributed within the SN (Zucca et al., 2014). NM normally accumulates with age in human SN neurons (Zecca et al., 2002). A neuronal pigment has also been observed in the SN of adult rats (Rabey and Hefti, 1990), and its concentration seems to depend upon age (DeMattei et al., 1986). Indeed, DA is one of the precursors in NM synthesis. NM is synthesized *via* iron-dependent oxidation of cytosolic catecholamines and subsequent reaction with proteins and lipids in midbrain DA neurons (Karlsson and Lindquist, 2013). NM biosynthesis is driven by an excess of cytosolic DA that not collected in synaptic vesicles (Sulzer et al., 2000). Excess cytosolic DA can be removed by converting it into a stable compound such as NM. The main iron compound in DA and norepinephrine neurons is the NM-iron complex, since NM is an effective metal chelator. NM serves to trap iron and provides neuronal protection from oxidative stress (Zucca et al., 2017). The biological function of NM is controversial. On one hand, NM plays a neuroprotective role in physiological states by combining with metal ions or drugs (Zecca et al., 2004). On the other hand, NM interacts with excessive Fe3 + and α-synuclein in the cytoplasm, damages Ca2 + homeostasis, and overactivates microglia, resulting in toxic effects and a pathological state (Zecca et al., 2008; Kim et al., 2020). When NM is combined with iron or other metals, other paramagnetic complexes result in T1-shortening effects in neuromelanin-sensitive magnetic resonance imaging (NM-MRI) (Trujillo et al., 2017; Salzman et al., 2021). Brain tissue signal intensity is reduced, and NM-containing nuclei show high signal intensity due to shortened T1 relaxation times (van der Pluijm et al., 2021). Thus, NM-MRI can enhance the T1-related contrast between NM and the brain tissue (Wengler et al., 2020), which allows NM-MRI to be used to detect NM accumulation. As NM is located in the cell bodies of dopaminergic neurons of the nigrostriatal pathway (Reneman et al., 2021), NM-MRI can also be used as a proxy measure for DA function. It provides the possibility for indirect measurement of DA function in non-neurodegenerative conditions.

Recent studies have suggested that NM-MRI may provide a complementary non-invasive proxy measure of DA function (Sulzer et al., 2018; Cassidy et al., 2019; Martín-Bastida et al., 2019). Interindividual variability in DA function may result in varying levels of NM accumulation in the SN, which can be detected by NM-MRI (Cassidy et al., 2019). Although the potential of NM-MRI as a candidate biomarker for dopaminergic pathology in patients with neurodegenerative disorders like PD has been demonstrated, studies in patients with non-neurodegenerative diseases are sparse. Critically, NM-MRI can reliably capture not only NM depletion in the SN in those with neurodegeneration disorders (Reimão et al., 2015; Fabbri et al., 2017) but also alterations in DA function in those without neurodegeneration (Shibata et al., 2008; Jauhar et al., 2017; Cassidy et al., 2019), based on *in vitro* evidence that stimulating DA synthesis boosts NM synthesis (Rice et al., 2016; Ashok et al., 2017). In addition, research on addiction and psychosis-related disease has shown that NM-MRI can be used to capture increases in NM accumulation in the SN (Weinstein et al., 2017; Cassidy et al., 2020).

In this study, we aimed to investigate the feasibility of using NM-MRI to measure NM concentrations in rats following acute MA administration and to explore whether NM-MRI signal intensity can be used as potential biomarker of DA function in non-neurodegenerative diseases.

## Materials and Methods

### Animals

Male Sprague–Dawley (SD) rats (180–220 g upon arrival) were purchased from Hunan Slack animal company (Changsha, China). The rats were housed in single cages in clean animal room on a 12-h light-dark cycle, with air humidity of 50–70% and room temperature controlled at 22–24°C. The experiment began after 7 days of adaptation to the environment. The weight of the rats before intubation surgery was up to 280–320 g, and the rats were reared in single cages after surgery. To control body weight levels, the rats were given 18–20 g of food daily during the experiment and had *ad libitum* access to water. Experimental protocols involving the animals were in accordance with the Guiding Opinions on Treating Experimental Animals well issued by the Ministry of Science and Technology of China in 2006. All experiments were conducted after obtaining approval from the institutional review board.

### Drug Treatment

A total of 27 rats were included in this study and divided into the experimental group (*n* = 16) and control group (*n* = 11). Intravenous administration was chosen because this route of administration is consistent with the most common administration methods in individuals who use MA, and the measurement in animal models is accurate and controllable (Cruickshank and Dyer, 2009). Intravenous administration delivers a higher concentration of the drug to the brain tissue than intraperitoneal injection. All subjects were surgically treated with jugular vein catheterization before administration. The intravenous intubation was ensured to be patent in each rat, and the rats with blocked catheters were eliminated. To prevent motion artifacts during MRI examinations, all subjects were anesthetized by an intraperitoneal injection of 30 mg/kg pentobarbital sodium solution (3%) and 0.2 ml atropine sulfate solution (0.5 mg/ml) before the MRI scan. Then, subjects in the experimental group were injected with MA (0.2 mg/kg of body weight; 0.2 mg/ml concentration of injected solution), and those in the control group were injected with placebo (0.9% normal saline; 0.2 mg/ml concentration of injected solution) through the jugular vein catheterization in their home cages. MRI scans were performed before and after acute drug injection. Because the amount of anesthesia required for the scan may have a significant impact on the rats’ survival, the experiment excluded rats that died from an anesthetic overdose or woke up midway through the scan with a low dose of anesthesia. Basic materials used in the experimental group and control group are shown in Table 1.

**TABLE 1:** Basic materials used in the experimental group and control group.

Group	Subject	Weight/g	Anesthetic (μl)	MA (μl)	Normal saline (μl)
Experimental group	1	380	380	380	—
	2	311	311	311	—
	3	323	323	323	—
	4	318	318	318	—
	5	291	291	291	—
	6	305	305	305	—
	7	292	292	292	—
	8	400	400	400	—
	9	412	412	412	—
	10	383	383	383	—
Control group	1	413	413	—	413
	2	395	395	—	395
	3	428	428	—	428
	4	403	403	—	403
	5	410	410	—	410
	6	343	343	—	343
	7	389	389	—	389
	8	405	405	—	405

*MA: 0.2 mg/kg of body weight, concentration of 0.2 mg/ml.*

*Anesthetic (3% pentobarbital sodium): 30 mg/kg of body weight.*

*Normal saline: 0.2 ml/kg of body weight.*

### Magnetic Resonance Imaging Acquisition

Magnetic resonance imaging scans were performed on a 3.0 T MRI system (μMR 790, United Imaging Healthcare, Shanghai, China) with a 12-channel rat coil. The study protocol included the following sequences: (1) 3D T1-weighted gradient-recalled echo (GRE) sequence [repetition time (TR)/echo time (TE) = 10.16/4.6 ms, flip angle (FA) = 12°, 26 slices, slice thickness = 1.2 mm, field of view (FOV) = 50 mm × 72 mm, and matrix = 167 × 240]; (2) T2-weighted fast spin echo (FSE) sequence (TR/TE = 6372/103.36 ms, FA = 145°, 26 slices, slice thickness = 1.2 mm, FOV = 50 mm × 72 mm, and matrix = 250 × 360); (3) NM-MRI sequence. NM-MRI was performed by using the GRE sequence with magnetization transfer (MT) pulse: TR/TE = 62/5.2 ms, FA = 40°, 6 slices, slice thickness = 1.2 mm, FOV = 50 mm × 72 mm, matrix = 167 × 240, MT frequency offset = 2,000 Hz, and duration = 10 ms. This protocol provided coverage of SN-containing portions of the midbrain and surrounding structures.

**FIGURE 1:**
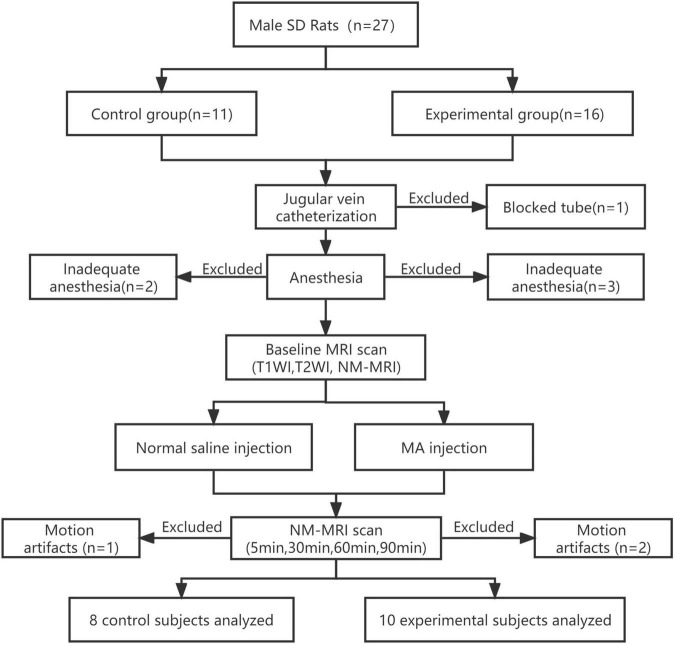
Experimental flow chart. MRI data from the experimental group (*n* = 10) and the control group (*n* = 8) were finally analyzed. SD, Sprague–Dawley; MA, methamphetamine; NM, neuromelanin; NM-MRI, neuromelanin-sensitive magnetic resonance imaging; T1WI, T1-weighted imaging; T2WI, T2-weighted imaging.

The NM-MRI scan before the drug injection was defined as the baseline scan. From the baseline NM-MRI scan before drug injection, T1-weighted images and T2-weighted images were acquired from all rats. One acute intrajugular injection of MA and normal saline were given after baseline NM-MRI scanning in the experimental group and control group, respectively. MA can quickly pass through the blood-brain barrier after entering the blood and takes approximately 5 min to act in the brain tissue. The peak time is approximately half an hour, and the concentration decreases after approximately an hour (Zhang et al., 2001). Thus, NM-MRI scans were performed 5, 30, 60 and 90 min after acute drug injection. The MRI room temperature was maintained at 22–24°C during the scan. The rats were wrapped in blankets to prevent hypothermia. The quality of NM-MRI images was visually inspected for artifacts immediately after acquisition by two experienced radiologists. Images of rats with severe motion artifacts affecting the midbrain or incorrect image-stack placement during scanning were excluded. One rat with a blocked catheter, three rats with inadequate anesthesia, and two rats that woke up midway through the procedure (and had severe motion artifacts affecting the midbrain) were excluded from the experimental group. Two rat with inadequate anesthesia and one rat with motion artifacts in the control group were excluded. Finally, MRI data from 10 rats in the experimental group and 8 rats in the control group were analyzed. All rats were euthanized after MRI scanning. The experimental flow chart is shown in Figure 1.

### Image Analysis

**FIGURE 2:**
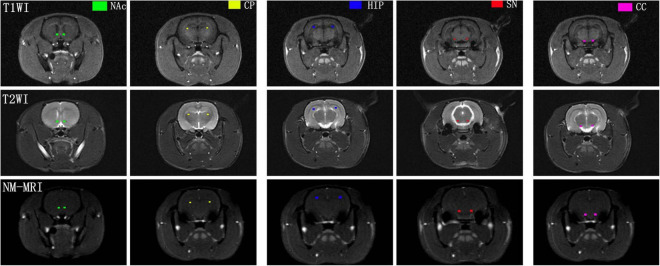
Representative NM-MRI images of brain regions of interest. CP, caudate putamen; NAc, nucleus accumbens; HIP, hippocampus; SN, substantia nigra; CC, crus cerebri.

To show the signal intensity changes in NM-MRI before and after acute MA administration, regions of interest (ROIs) in each subject, which included the SN, CP, NAc, and hippocampus (HIP) were manually drawn by one experienced radiologist (10 years of experience in brain MRI interpretation) with reference to a brain atlas (Marinković et al., 2020). The crus cerebri (CC) is a white matter tract known to have minimal NM content and was drawn as a reference region. This approach captures topographic alterations presumably corresponding with DA neurons that are functionally altered in drug addiction, and this approach was previously shown to have high sensitivity to dopaminergic pathophysiology (Cassidy et al., 2019). All the ROIs were first delineated on the T1-weighted image and then transferred to the NM-MRI images. Mean values of NM-MRI signal intensity in the bilateral ROIs at different time points were recorded. Representative images of brain regions are shown in Figure 2.

### Statistical Analysis

All statistical analyses were conducted using SPSS Statistics version 22 (IBM, NY, United States). Rat body weights, anesthesia, and MA dose for all subjects are listed in Table 1. To exclude irrelevant variables, a series of Pearson correlation analyses were adopted to evaluate the relationships between NM-MRI signal intensity and weight, anesthesia, and MA dose for each subject. For each of the five brain regions, the mean values of the NM-MRI signal intensity at different time points of both groups were compared using ANOVA. In addition, the average signal intensity values in different brain regions at the same time point were calculated and compared using ANOVA. Significance was at the 95% confidence level. Multiple comparison correction was performed by the false discovery rate (FDR) method.

## Results

### Neuromelanin-Sensitive-Magnetic Resonance Imaging Signal Intensity in the Substantia Nigra Was Higher Than in Other Brain Regions

**FIGURE 3:**
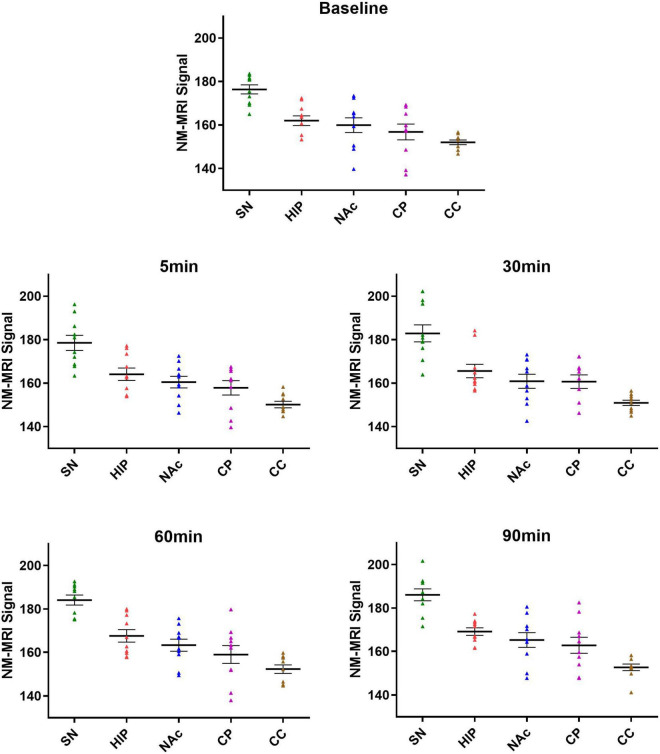
Average signal intensities in each brain region at different time points in the experimental group. At all the time points (baseline and 5, 30, 60, and 90 min), the lowest to highest NM-MRI signal intensity across brain regions were observed in the CC, CP, NAc, HIP and SN. NM-MRI signal intensities in the SN were significantly higher than those in other brain regions at each time point (*P* < 0.05).

There was no significant correlation between the NM-MRI signal intensity after MA administration and the rat weight, anesthesia, and MA dose in the experimental and control groups. The average signal intensity values in the bilateral SN, HIP, NAc, CP and CC in the experimental and control groups are shown in Table 2. As shown in Figure 3, at each time point (baseline and 5, 30, 60, and 90 min), lowest to highest NM-MRI signal intensity were detected in the CC, CP, NAc, HIP and SN in both the experimental and control groups. The SN had the highest signal intensity, while the CC had the lowest signal intensity. In addition, NM-MRI signal intensity values in the SN were significantly higher than those in the other brain regions at each time point in both the experimental (*P* < 0.05) and control (*P* < 0.05) groups.

**TABLE 2:** Average NM-MRI signal intensities in different brain regions at each time point (mean ± SD).

Time point		Baseline	5 min	30 min	60 min	90 min
Experimental group	SN	176.38 ± 6.64	178.55 ± 11.08	182.93 ± 12.41	184.13 ± 7.27	186.10 ± 8.60
	HIP	162.03 ± 7.07	164.10 ± 9.12	165.61 ± 9.81	167.58 ± 9.07	169.10 ± 5.42
	NAc	159.93 ± 10.71	160.51 ± 8.51	160.87 ± 10.18	163.27 ± 8.75	165.21 ± 10.76
	CP	156.81 ± 11.55	157.87 ± 10.41	160.68 ± 9.93	158.98 ± 12.95	162.80 ± 11.78
	CC	152.05 ± 3.33	150.18 ± 4.75	150.98 ± 3.86	152.30 ± 6.23	152.66 ± 4.72
Control group	SN	174.45 ± 6.83	176.49 ± 7.46	176.76 ± 5.43	176.50 ± 3.97	177.33 ± 3.34
	HIP	163.51 ± 4.10	165.12 ± 4.51	164.91 ± 6.38	165.79 ± 8.19	167.15 ± 4.87
	NAc	160.36 ± 3.24	160.72 ± 3.51	160.58 ± 6.11	162.93 ± 3.84	162.89 ± 6.60
	CP	157.89 ± 4.20	158.99 ± 3.05	159.70 ± 4.75	158.82 ± 3.37	160.57 ± 3.50
	CC	151.38 ± 2.03	150.66 ± 3.65	150.86 ± 1.86	150.86 ± 2.65	152.15 ± 6.16

*The signal intensities are displayed as mean ± standard deviation.*

*NM-MRI signal intensities in the SN were significantly higher than those in other brain regions at each time point in both the experimental group (P < 0.05) and control group (P < 0.05).*

### Neuromelanin-Sensitive-Magnetic Resonance Imaging Signal Intensity in Each Brain Region Showed a Continuous Upward Trend After Acute Methamphetamine Administration

**FIGURE 4:**
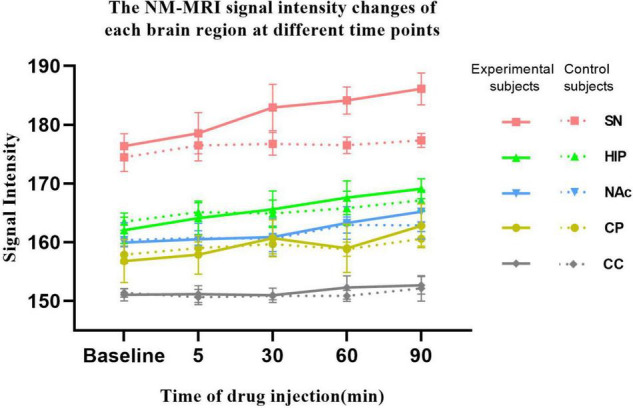
NM-MRI signal intensity changes in each brain region at different time points. The solid line represents the experimental group (*n* = 10), and the dotted line represents the control group (*n* = 8). NM-MRI signal intensities in addiction-related brain regions (SN, HIP, NAc, and CP) showed upward trends after acute MA administration, but no significant changes were observed in the control group. The NM-MRI signal intensity of SN in the experimental group was statistically significant higher at the 60 and 90 min compared with that in the control group (*P* values were 0.042 and 0.042, respectively, with FDR correction). In the experimental group, the NM-MRI signal intensity of SN was statistically significant higher at the 60 and 90 min compared with that before MA administration (*P* values were 0.023 and 0.011 respectively).

As shown in Figure 4, in the experimental group, acute MA administration resulted in continuous upward trends in NM-MRI signal intensity in the SN, HIP, and NAc over time, but there was no significant change in the CC. Although the magnitude of the increase varied between brain regions, signal intensity in all brain regions peaked at 90 min. After MA administration, signal intensity in the SN increased from 176.38 at baseline to 186.10 at 90 min, signal intensity in the HIP increased from 162.02 to 169.10, and signal intensity in the NAc increased from 159.93 to 165.20. However, signal intensity in the CP increased gradually at 5 and 30 min after MA administration, decreased slightly at 60 min, and finally increased at 90 min to reach its highest value. In contrast, in control group, signal intensities in all the brain regions did not significantly change over time after injection of normal saline. To assess a potential difference between the experimental group and the control group due to MA injection, we assessed the statistical difference in signal intensity between the two groups at each time point using ANOVA. The results showed that the NM-MRI signal intensity of SN in the experimental group was significantly higher at the 60 and 90 min compared with that in the control group (*P* values were 0.042 and 0.042, respectively, with FDR correction). In addition, in the experimental group, the NM-MRI signal intensity of SN was statistically significant higher at the 60 and 90 min compared with that before MA administration (*P* values were 0.023 and 0.011 respectively). In addition to the SN, the NM-MRI signal intensity in other brain regions were not statistically significant at any time point. In the control group, there was no statistical significance in each brain region at any time point.

**FIGURE 5:**
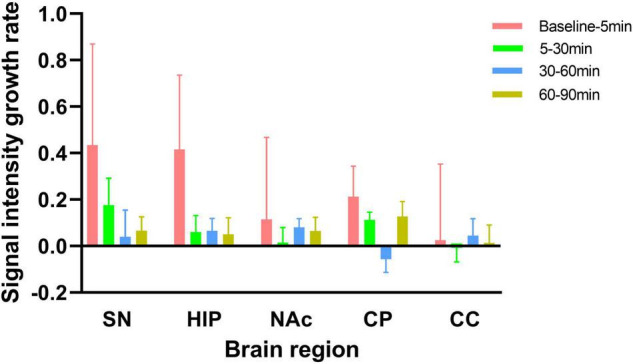
Signal intensity growth rates from baseline to 5 min, from 5 to 30 min, from 30 to 60 min, and from 60 to 90 min in the experimental group. The signal intensity growth rate is calculated by dividing the increase signal intensity by time. NM-MRI signal intensity growth rates in each brain region were the highest from baseline to 5 min after acute MA administration compared with other time periods.

As shown in Figure 5, from baseline to 5 min, from 5 to 30 min, from 30 to 60 min, and from 60 to 90 min after MA administration in experimental group, the growth rates in the SN signal intensity were 0.43, 0.18, 0.04, and 0.07, respectively; those in the HIP were 0.42, 0.06, 0.07, and 0.05, respectively; those in the NAc were 0.11, 0.01, 0.08, and 0.06, respectively; and those in the CP were 0.21, 0.11, −0.06, and 0.13, respectively. However, there were no significant increases in CC signal intensity across any of these time periods. Although no significant differences were found, the growth rates in the NM-MRI signal intensities in the SN, HIP, NAc and CP were the highest within 5 min after acute MA administration compared with other time periods. In contrast, during the periods from 5–30 min, 30–60 min, and 60–90 min, the growth rates decreased to varying degrees between brain regions.

### Increased Amplitude and Rate of Change in Neuromelanin-Sensitive-Magnetic Resonance Imaging Signal Intensity in the Substantia Nigra Were Higher Than That in Other Brain Regions After Acute Methamphetamine Administration

**FIGURE 6:**
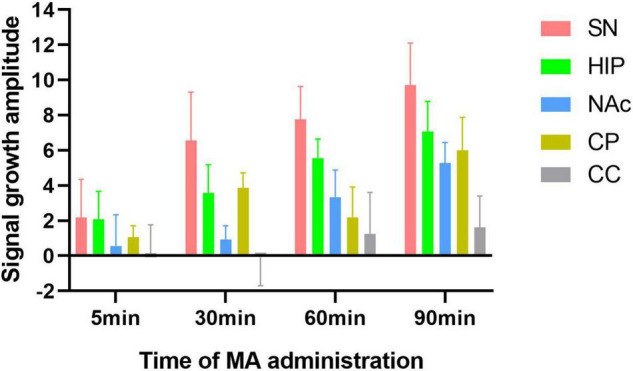
Amplitude of signal growth at four time points after MA administration compared to baseline in the experimental group. The increased amplitudes in NM-MRI signal intensity in the SN were higher than those in other brain regions at each time point compared to baseline after acute MA administration.

Increases in signal intensity in different brain regions at different time points compared to baseline were calculated. In the experimental group, the NM-MRI signal intensity at 5 min after MA administration, compared with baseline, in the SN, HIP, CP, and NAc increased by 2.17, 2.08, 1.06, and 0.57, respectively. At 30 min compared with baseline, NM-MRI signal intensity in the SN, HIP, CP and NAc increased by 6.55, 3.58, 3.87 and 0.93, respectively. At 60 min compared with baseline, NM-MRI signal intensities in the SN, HIP, CP and NAc increased by 7.75, 5.55, 2.18 and 3.34, respectively. At 90 min compared with baseline, NM-MRI signal intensities in the SN, HIP, CP and NAc increased by 9.73, 7.08, 5.99 and 5.27, respectively. There were no significant changes in the CC over time. As shown in Figure 6, although there was no statistically significant difference, the increased amplitudes in the NM-MRI signal intensities in the SN were higher than those in other brain regions at each time point after acute MA administration compared to baseline. In addition, the increased rates of change in the NM-MRI signal intensity in the SN were also higher than those in other brain regions. Statistically, the NM-MRI signal intensity in the SN increased significantly more at 30 min than in the NAc (*P* < 0.05). The NM-MRI signal intensity in the SN increased significantly more at 60 min than in the CP (*P* < 0.05).

## Discussion

The present study assessed the effect of acute MA exposure on functional changes in dopaminergic pathways by NM-MRI. The results demonstrated that the NM-MRI signal intensity in the SN was significantly higher than that in other brain regions at all the time points. This is consistent with the fact that NM is mainly deposited in the SN as presented in a previous study (Zecca et al., 2001). This study further demonstrated that NM-MRI is sensitive enough to detect regional variability in concentrations of NM in brain tissue, which presumably depends on interindividual and interregional differences in DA function (Poulin et al., 2020). NM-MRI has the ability to directly demonstrate different signal intensities based on the NM content of their constituent neurons. This is a prerequisite for its use as a biomarker of interindividual variability in DA function following MA exposure. Since the results indicated that NM-MRI signal intensity corresponded to regional tissue concentration of NM, particularly in the midbrain region of the SN, NM accumulation probably correlated with higher DA release from nigrostriatal SN neurons. Together, these findings open the possibility that NM-MRI could be used as a proxy measure assessing DA function.

Interestingly, this study found that the lowest to highest NM-MRI signal intensity across brain regions were observed in the CC, CP, NAc, HIP and SN before and after MA administration. The SN had the highest signal intensity compared to other brain regions at each time point. NM has been shown to be richest in the SN but it is practically ubiquitous throughout the brain as it is diffusely distributed in smaller amounts in neurons of other brain areas (Zecca et al., 2008; Ward et al., 2009). Our results suggested that the deposition of NM in the CC, CP, NAc, HIP and SN, which showed a gradual increase from low to high, with highest levels in the SN and lowest levels in the CC. These results probably related to the cellular heterogeneity of midbrain dopaminergic neurons (La Manno et al., 2016), and NM-MRI has the ability to detect regional heterogeneity based on NM content. Interindividual variability in DA function may result in varying levels of NM accumulation in the SN (Cassidy et al., 2019). Therefore, the differences in NM content across brain regions may be related to the diverse array of DA functions. The heterogeneity in the cell populations in the midbrain suggests that DA function may differ substantially between neuronal tiers projecting to other regions (Joel and Weiner, 2000; Rice et al., 2016; Weinstein et al., 2017). Moreover, according to prior research, the SN contains the vast majority of DA neurons among the traditionally defined midbrain DA groups (Anderegg et al., 2015),which is consistent with higher signal intensity in the SN in our study.

Having shown that NM-MRI measures the disparity in regional concentrations of NM in the SN and other brain regions, we then focused on evaluating the signal intensity changes in different brain regions over time after acute MA administration, to test whether increased NM-MRI signal intensities correlated with DA function *in vivo*. By analyzing the signal intensity changes at different time points after acute MA administration, the results showed that acute MA administration contributed to a continuous upward trend in NM-MRI signal intensities in four brain regions, all regions except for the CC, from baseline to 90 min, although the magnitude of increase varied across brain regions. However, this upward trend in signal intensity was not seen in the control group following the normal saline injection. These results suggested that MA administration activated the mesolimbic (NAc and HIP) and nigrostriatal (SN and CP) pathways, and enhanced DA release at the dopaminergic terminals, leading to an increase in NM-MRI signal intensity in addiction-related brain regions. One interpretation is that MA exposure is associated with a redistribution of DA between cytosolic and vesicular pools (Cassidy et al., 2020). MA inhibits vesicular monoamine transporter 2 (VMAT2) and promotes DA release from synaptic vesicles into the cytoplasm. Subsequently, available cytosolic DA is reverse transported by DATs into the extracellular space, resulting ultimately in increased extracellular DA (Fleckenstein et al., 2007; Skinbjerg et al., 2010; Nickell et al., 2014). A decrease in VMAT2 could account for the increased NM-MRI signal intensity; that is, decreased VMAT2 expression would decrease vesicular DA and increase the cytosolic DA pool from which NM is synthesized (Sulzer et al., 2000; Markov et al., 2008; Zucca et al., 2014). Excess cytosolic DA is thought to copolymerize with cysteine into a polymer of ill-defined composition which is eventually packaged into larger double-membrane bound vesicles (NM granules), leading to increased accumulation of NM (Haining and Achat-Mendes, 2017). Sulzer et al. (2000) showed that NM forms in cultured dopaminergic neurons when cytoplasmic concentrations of DA are artificially increased. Another explanation is that NM synthesis itself acts as a protective mechanism, clearing out excess cytoplasmic catecholamines and increasing NM synthesis (Zecca et al., 2002). Oxidative stress is necessitated for the handling of high concentrations of catecholamines, while the protective role of NM in dopaminergic neurons lies in its prevention of neurotoxicity from quinones that are formed during DA oxidation. When DA is oxidized to dopamine o-quinone, aminochrome and 5,6-indolequinone are formed and typically undergo polymerization to form the dark pigment NM (Muñoz et al., 2012b). The product of oxidative stress (i.e., NM) could then function to alleviate the very thing that created itself (i.e., excess DA). As has been pointed out, sequestration into vesicles apparently alleviates this toxicity and stress on the cell. This process suggests that the accumulation of NM itself could serve as a storage, protection, and rerelease mechanism for DA, possibly acting as an actual molecular memory loop. This could partly explain the reinforcement of addictive memories and behavior that are associated with DA-releasing drugs. Previous studies have shown that PD is characterized by the loss of DA neurons, resulting in decreased NM concentration and NM-MRI signal intensity (Sasaki et al., 2006; Sulzer et al., 2018; Martín-Bastida et al., 2019). However, MA exposure produces the opposite effects. Acute MA exposure is characterized by increased DA concentrations in most brain regions (Czoty et al., 2004; Desai et al., 2010), and our results indicated that the increased NM-MRI signal intensity may have been due to increased NM concentrations, which may indirectly reflect the elevated DA availability in DA neurons and is consistent with the idea that NM accumulation depends on DA function in the soma (Sulzer et al., 2000; Cebrián et al., 2014; Cassidy et al., 2019). Thus, our results supported the idea that NM-MRI captures an addiction-related aspect of DA dysfunction in the nigrostriatal pathway.

In this study, across each of the brain regions, the signal intensity growth rate within 5 min of acute MA administration was the highest compared with other time periods, which indicated that DA release in the four brain regions occurred most rapidly within 5 min after acute MA administration compared with other time periods. A previous study showed that extracellular DA concentrations in the SN, CP and NAc increased with a peak response at 30 min after acute MA injections (Zhang et al., 2001). One possible explanation for the current results was the delay in scanning. In particular, it took 20 min to complete the NM-MRI scan after the MA injection. Another explanation is that MA was administered differently in this study. Intraperitoneal injections were used in a previous study (Zhang et al., 2001), while jugular vein injections were used in this study, a route by which the drug has a faster effect.

Having validated that the signal intensity increased in most brain regions over time, we then proceeded to analyze the differences in increased signal intensities in different brain regions at different time points. In this study, the NM-MRI signal intensity of SN was significantly higher at the 60 and 90 min both in within-groups and inter-group comparison. There was no significant result in other brain regions, which suggested that a significant effect from MA administration in SN. The rate of increase and amplitude of NM-MRI signal intensities in the SN were higher than those in other brain regions at each time point after acute MA administration. These results suggested that the conversion of DA to NM was most significant in the SN after acute MA administration, which was consistent with the SN being where most NM accumulates. Given that a previous imaging study showed that cocaine users had higher locus coeruleus (LC) NM signal intensity but the VTA/SN did not show significant group differences (Wang et al., 2021), the finding of NM-MRI signal intensity increases being most significant in the SN provides additional insight into the pathophysiology of MA exposure. The combination of increased NM-MRI signal intensity and elevated NM in the SN suggests that DA neurons are differently distributed after MA exposure. The SN is DA-rich and contains both redox-available NM and a high iron content (Zucca et al., 2014). There are several interrelated mechanisms involving iron, DA, and NM in SN neurons (Zucca et al., 2017). NM accumulates during aging and is the catecholamine-derived pigment in the dopaminergic neurons in the SN, but it is relatively depleted in the SN of patients with PD. On one hand, NM is an effective metal chelator and serves to trap iron and provides neuronal protection from oxidative stress. The high concentration of NM in SN neurons seems to be linked to the presence of a considerable amount of cytosolic DA that has not been sequestered into synaptic vesicles. Excess cytosolic DA can be removed by converting it into a stable compound like NM, and this process rescues the cell, that is, NM serves a protective role (Muñoz et al., 2012a). Our results could be explained by this neuroprotective effect in that NM synthesis was most significant in the SN. On the other hand, many cellular redox reactions rely on iron, however, an altered distribution of reactive iron is cytotoxic (Wang et al., 2021).

In this study, we first demonstrated that NM-MRI is sensitive enough to detect regional heterogeneity in NM content and found that the SN itself accumulates the most NM. Secondly, the NM-MRI signal intensity in each brain region showed an upward trend after MA administration. consistent with the evidence that DA dysfunction is closely related to drug addiction, and thus, our data support the notion that the increase in DA concentration would lead to increases in NM deposition over time, which can be captured by NM-MRI. Finally, the conversion of DA to NM was most significant in the SN, which also confirmed that NM is mainly deposited in the SN. Our work suggested that NM-MRI has the ability to capture this addiction-related phenotype consisting of nigrostriatal DA excess by demonstrating an increase in NM accumulation in the SN. These findings promote the development of this candidate biomarker for addiction-related psychiatric disorders and treatment selection.

There are several limitations in this study. First, this study lacked an absolute gold standard. It is critical that future studies should provide direct evidence for a relationship between NM-MRI signal intensity and concentration of DA and NM measured in *ex vivo* rat brain tissue using biological methods. Second, similar to other neuropsychiatric diseases, our results showed that the NM-MRI signal intensity is sensitive but not necessarily diagnosis-specific for MA addiction; applications in other non-degenerative diseases remain to be studied. Third, there was no monitoring of physiological parameters during the experiment and that a potential bias due to a change in the animal’s body temperature cannot be excluded. Finally, our study was limited to animals and needs to be validated in the context of a clinical application in the future.

## Conclusion

This study was an investigation to identify evidence for abnormal NM-MRI signal intensity in NM-containing dopaminergic neurons in the SN after acute MA exposure. Given the central role of DA in drug addiction and the non-invasiveness of NM-MRI data acquisition, NM-MRI has the potential to advance our understanding of DA alterations in the context of drug addiction. Our findings further underscore the promise of NM-MRI as a method to identify clinically useful biomarkers for non-neurodegenerative conditions associated with DA dysfunction.

## Data Availability Statement

The original contributions presented in the study are included in the article/supplementary material, further inquiries can be directed to the corresponding author/s.

## Ethics Statement

The animal study was reviewed and approved by the institutional review board in the Second Xiangya Hospital of Central South University.

## Author Contributions

JL and FT: conception and design. JL and HL: administrative support. FT, XZ, and HZ: collection and data assembly. JL, FT, WY, YMD, YYD, and LZ: data analysis and interpretation. All authors wrote the manuscript and approved the manuscript.

## Conflict of Interest

YMD and LZ were employed by the company Shanghai United Imaging Healthcare Co., Ltd. The remaining authors declare that the research was conducted in the absence of any commercial or financial relationships that could be construed as a potential conflict of interest.

## Publisher’s Note

All claims expressed in this article are solely those of the authors and do not necessarily represent those of their affiliated organizations, or those of the publisher, the editors and the reviewers. Any product that may be evaluated in this article, or claim that may be made by its manufacturer, is not guaranteed or endorsed by the publisher.

## References

[B1] Anderegg A. Poulin J. F. Awatramani R. (2015). Molecular heterogeneity of midbrain dopaminergic neurons–Moving toward single cell resolution. *FEBS Lett.* 589 3714–3726. 10.1016/j.febslet.2015.10.022 26505674 PMC4679573

[B2] Ares-Santos S. Granado N. Espadas I. Martinez-Murillo R. Moratalla R. (2014). Methamphetamine causes degeneration of dopamine cell bodies and terminals of the nigrostriatal pathway evidenced by silver staining. *Neuropsychopharmacology* 39 1066–1080. 10.1038/npp.2013.307 24169803 PMC3957101

[B3] Ashok A. H. Mizuno Y. Volkow N. D. Howes O. D. (2017). Association of Stimulant Use With Dopaminergic Alterations in Users of Cocaine, Amphetamine, or Methamphetamine: A Systematic Review and Meta-analysis. *JAMA Psychiatry* 74 511–519. 10.1001/jamapsychiatry.2017.0135 28297025 PMC5419581

[B4] Björklund A. Dunnett S. B. (2007). Dopamine neuron systems in the brain: an update. *Trends Neurosci.* 30 194–202. 10.1016/j.tins.2007.03.006 17408759

[B5] Bubenikova-Valesova V. Kacer P. Syslova K. Rambousek L. Janovsky M. Schutova B. (2009). Prenatal methamphetamine exposure affects the mesolimbic dopaminergic system and behavior in adult offspring. *Int. J. Dev. Neurosci.* 27 525–530. 10.1016/j.ijdevneu.2009.06.012 19591914

[B6] Cassidy C. M. Carpenter K. M. Konova A. B. Cheung V. Grassetti A. Zecca L. (2020). Evidence for Dopamine Abnormalities in the Substantia Nigra in Cocaine Addiction Revealed by Neuromelanin-Sensitive MRI. *Am. J. Psychiatry* 177 1038–1047. 10.1176/appi.ajp.2020.20010090 32854531 PMC9108998

[B7] Cassidy C. M. Zucca F. A. Girgis R. R. Baker S. C. Weinstein J. J. Sharp M. E. (2019). Neuromelanin-sensitive MRI as a noninvasive proxy measure of dopamine function in the human brain. *Proc. Natl. Acad. Sci. U.S.A.* 116 5108–5117. 10.1073/pnas.1807983116 30796187 PMC6421437

[B8] Cebrián C. Zucca F. A. Mauri P. Steinbeck J. A. Studer L. Scherzer C. R. (2014). MHC-I expression renders catecholaminergic neurons susceptible to T-cell-mediated degeneration. *Nat. Commun.* 5:3633. 10.1038/ncomms4633 24736453 PMC4024461

[B9] Cruickshank C. C. Dyer K. R. (2009). A review of the clinical pharmacology of methamphetamine. *Addiction* 104 1085–1099. 10.1111/j.1360-0443.2009.02564.x 19426289

[B10] Czoty P. W. Makriyannis A. Bergman J. (2004). Methamphetamine discrimination and in vivo microdialysis in squirrel monkeys. *Psychopharmacology* 175 170–178. 10.1007/s00213-004-1798-6 15064912

[B11] DeMattei M. Levi A. C. Fariello R. G. (1986). Neuromelanic pigment in substantia nigra neurons of rats and dogs. *Neurosci. Lett.* 72 37–42. 10.1016/0304-3940(86)90614-2 3492690

[B12] Desai R. I. Paronis C. A. Martin J. Desai R. Bergman J. (2010). Monoaminergic psychomotor stimulants: discriminative stimulus effects and dopamine efflux. *J. Pharmacol. Exp. Ther.* 333 834–843. 10.1124/jpet.110.165746 20190012 PMC2879939

[B13] Fabbri M. Reimão S. Carvalho M. Nunes R. G. Abreu D. Guedes L. C. (2017). Substantia Nigra Neuromelanin as an Imaging Biomarker of Disease Progression in Parkinson’s Disease. *J. Park. Dis.* 7 491–501. 10.3233/JPD-171135 28671143

[B14] Fleckenstein A. E. Volz T. J. Riddle E. L. Gibb J. W. Hanson G. R. (2007). New insights into the mechanism of action of amphetamines. *Annu. Rev. Pharmacol. Toxicol.* 47 681–698. 10.1146/annurev.pharmtox.47.120505.105140 17209801

[B15] Granado N. Ares-Santos S. O’Shea E. Vicario-Abejón C. Colado M. I. Moratalla R. (2010). Selective vulnerability in striosomes and in the nigrostriatal dopaminergic pathway after methamphetamine administration : early loss of TH in striosomes after methamphetamine. *Neurotox. Res.* 18 48–58. 10.1007/s12640-009-9106-1 19760475 PMC2875475

[B16] Granado N. Ares-Santos S. Tizabi Y. Moratalla R. (2018). Striatal Reinnervation Process after Acute Methamphetamine-Induced Dopaminergic Degeneration in Mice. *Neurotox. Res.* 34 627–639. 10.1007/s12640-018-9925-z 29934756

[B17] Groman S. M. Lee B. Seu E. James A. S. Feiler K. Mandelkern M. A. (2012). Dysregulation of D_2_-mediated dopamine transmission in monkeys after chronic escalating methamphetamine exposure. *J. Neurosci.* 32 5843–5852. 10.1523/JNEUROSCI.0029-12.2012 22539846 PMC3353813

[B18] Haining R. L. Achat-Mendes C. (2017). Neuromelanin, one of the most overlooked molecules in modern medicine, is not a spectator. *Neural Regen. Res.* 12 372–375. 10.4103/1673-5374.202928 28469642 PMC5399705

[B19] Huang W. S. Chen G. J. Tsai T. H. Cheng C. Y. Shiue C. Y. Ma K. H. (2019). In vivo long-lasting alterations of central serotonin transporter activity and associated dopamine synthesis after acute repeated administration of methamphetamine. *EJNMMI Res.* 9:92. 10.1186/s13550-019-0557-y 31535286 PMC6751231

[B20] Jauhar S. Nour M. M. Veronese M. Rogdaki M. Bonoldi I. Azis M. (2017). A Test of the Transdiagnostic Dopamine Hypothesis of Psychosis Using Positron Emission Tomographic Imaging in Bipolar Affective Disorder and Schizophrenia. *JAMA Psychiatry* 74 1206–1213. 10.1001/jamapsychiatry.2017.2943 29049482 PMC6059355

[B21] Joel D. Weiner I. (2000). The connections of the dopaminergic system with the striatum in rats and primates: an analysis with respect to the functional and compartmental organization of the striatum. *Neuroscience* 96 451–474. 10.1016/s0306-4522(99)00575-8 10717427

[B22] Karlsson O. Lindquist N. G. (2013). Melanin affinity and its possible role in neurodegeneration. *J. Neural Trans.* 120 1623–1630. 10.1007/s00702-013-1062-5 23821370

[B23] Kim B. Yun J. Park B. (2020). Methamphetamine-Induced Neuronal Damage: neurotoxicity and Neuroinflammation. *Biomol. Ther.* 28 381–388. 10.4062/biomolther.2020.044 32668144 PMC7457172

[B24] La Manno G. Gyllborg D. Codeluppi S. Nishimura K. Salto C. Zeisel A. (2016). Molecular Diversity of Midbrain Development in Mouse, Human, and Stem Cells. *Cell* 167 566.e–580.e. 10.1016/j.cell.2016.09.027 27716510 PMC5055122

[B25] Lammel S. Lim B. K. Malenka R. C. (2014). Reward and aversion in a heterogeneous midbrain dopamine system. *Neuropharmacology* 76 351–359. 10.1016/j.neuropharm.2013.03.019 23578393 PMC3778102

[B26] Lin M. Sambo D. Khoshbouei H. (2016). Methamphetamine Regulation of Firing Activity of Dopamine Neurons. *J. Neurosci.* 36 10376–10391. 10.1523/JNEUROSCI.1392-16.2016 27707972 PMC5050330

[B27] Lu S. Yang Y. Liao L. Yan W. Xiong K. Yan J. (2021). iTRAQ-based proteomic analysis of the rat striatum in response to methamphetamine preconditioning. *Acta Biochim. Biophys. Sin.* 53 636–639. 10.1093/abbs/gmab024 33742667

[B28] Marinković I. Tatlisumak T. Abo-Ramadan U. Brkić B. G. Aksić M. Marinković S. (2020). A basic MRI anatomy of the rat brain in coronal sections for practical guidance to neuroscientists. *Brain Res.* 1747:147021. 10.1016/j.brainres.2020.147021 32755602

[B29] Markov D. Mosharov E. V. Setlik W. Gershon M. D. Sulzer D. (2008). Secretory vesicle rebound hyperacidification and increased quantal size resulting from prolonged methamphetamine exposure. *J. Neurochem.* 107 1709–1721. 10.1111/j.1471-4159.2008.05737.x 19014382 PMC3081719

[B30] Martín-Bastida A. Lao-Kaim N. P. Roussakis A. A. Searle G. E. Xing Y. Gunn R. N. (2019). Relationship between neuromelanin and dopamine terminals within the Parkinson’s nigrostriatal system. *Brain* 142 2023–2036. 10.1093/brain/awz120 31056699 PMC6664390

[B31] Moratalla R. Khairnar A. Simola N. Granado N. García-Montes J. R. Porceddu P. F. (2017). Amphetamine-related drugs neurotoxicity in humans and in experimental animals: main mechanisms. *Prog. Neurobiol.* 155 149–170. 10.1016/j.pneurobio.2015.09.011 26455459

[B32] Muñoz P. Huenchuguala S. Paris I. Cuevas C. Villa M. Caviedes P. (2012a). Protective effects of nicotine against aminochrome-induced toxicity in substantia nigra derived cells: implications for Parkinson’s disease. *Neurotox. Res.* 22 177–180. 10.1007/s12640-012-9326-7 22528249 PMC3671757

[B33] Muñoz P. Huenchuguala S. Paris I. Segura-Aguilar J. (2012b). Dopamine oxidation and autophagy. *Park. Dis.* 2012:920953. 10.1155/2012/920953 22966478 PMC3433151

[B34] Nickell J. R. Siripurapu K. B. Vartak A. Crooks P. A. Dwoskin L. P. (2014). The vesicular monoamine transporter-2: an important pharmacological target for the discovery of novel therapeutics to treat methamphetamine abuse. *Adv. Pharmacol.* 69 71–106. 10.1016/B978-0-12-420118-7.00002-0 24484975 PMC4084610

[B35] Poulin J. F. Gaertner Z. Moreno-Ramos O. A. Awatramani R. (2020). Classification of Midbrain Dopamine Neurons Using Single-Cell Gene Expression Profiling Approaches. *Trends Neurosci.* 43 155–169. 10.1016/j.tins.2020.01.004 32101709 PMC7285906

[B36] Rabey J. M. Hefti F. (1990). Neuromelanin synthesis in rat and human substantia nigra. *J. Neural Trans. Park. Dis. Dement. Sect.* 2 1–14. 10.1007/BF02251241 2357268

[B37] Reimão S. Pita Lobo P. Neutel D. Correia Guedes L. Coelho M. Rosa M. M. (2015). Substantia nigra neuromelanin magnetic resonance imaging in de novo Parkinson’s disease patients. *Eur. J. Neurol.* 22 540–546. 10.1111/ene.12613 25534480

[B38] Reneman L. van der Pluijm M. Schrantee A. van de Giessen E. (2021). Imaging of the dopamine system with focus on pharmacological MRI and neuromelanin imaging. *Eur. J. Radiol.* 140:109752. 10.1016/j.ejrad.2021.109752 34004428

[B39] Rice M. W. Roberts R. C. Melendez-Ferro M. Perez-Costas E. (2016). Mapping dopaminergic deficiencies in the substantia nigra/ventral tegmental area in schizophrenia. *Brain Struct. Funct.* 221 185–201. 10.1007/s00429-014-0901-y 25269834 PMC4504823

[B40] Saha K. Sambo D. Richardson B. D. Lin L. M. Butler B. Villarroel L. (2014). Intracellular methamphetamine prevents the dopamine-induced enhancement of neuronal firing. *J. Biol. Chem.* 289 22246–22257. 10.1074/jbc.M114.563056 24962577 PMC4139236

[B41] Salzman G. Kim J. Horga G. Wengler K. (2021). Standardized Data Acquisition for Neuromelanin-sensitive Magnetic Resonance Imaging of the Substantia Nigra. *J. Vis. Exp.* 175:e62493. 10.3791/62493 PMC9124347 34570093

[B42] Sasaki M. Shibata E. Tohyama K. Takahashi J. Otsuka K. Tsuchiya K. (2006). Neuromelanin magnetic resonance imaging of locus ceruleus and substantia nigra in Parkinson’s disease. *Neuroreport* 17 1215–1218. 10.1097/01.wnr.0000227984.84927.a7 16837857

[B43] Shi W. X. Pun C. L. Zhang X. X. Jones M. D. Bunney B. S. (2000). Dual effects of D-amphetamine on dopamine neurons mediated by dopamine and nondopamine receptors. *J. Neurosci.* 20 3504–3511. 10.1523/JNEUROSCI.20-09-03504.2000 10777813 PMC6773133

[B44] Shibata E. Sasaki M. Tohyama K. Otsuka K. Endoh J. Terayama Y. (2008). Use of neuromelanin-sensitive MRI to distinguish schizophrenic and depressive patients and healthy individuals based on signal alterations in the substantia nigra and locus ceruleus. *Biol. Psychiatry* 64 401–406. 10.1016/j.biopsych.2008.03.021 18452894

[B45] Skinbjerg M. Liow J. S. Seneca N. Hong J. Lu S. Thorsell A. (2010). D2 dopamine receptor internalization prolongs the decrease of radioligand binding after amphetamine: a PET study in a receptor internalization-deficient mouse model. *NeuroImage* 50 1402–1407. 10.1016/j.neuroimage.2010.01.055 20097293 PMC2838946

[B46] Sulzer D. Bogulavsky J. Larsen K. E. Behr G. Karatekin E. Kleinman M. H. (2000). Neuromelanin biosynthesis is driven by excess cytosolic catecholamines not accumulated by synaptic vesicles. *Proc. Natl. Acad. Sci. U.S.A.* 97 11869–11874. 10.1073/pnas.97.22.11869 11050221 PMC17261

[B47] Sulzer D. Cassidy C. Horga G. Kang U. J. Fahn S. Casella L. (2018). Neuromelanin detection by magnetic resonance imaging (MRI) and its promise as a biomarker for Parkinson’s disease. *NPJ Park. Dis.* 4:11. 10.1038/s41531-018-0047-3 29644335 PMC5893576

[B48] Thanos P. K. Kim R. Delis F. Ananth M. Chachati G. Rocco M. J. (2016). Chronic Methamphetamine Effects on Brain Structure and Function in Rats. *PLoS One* 11:e0155457. 10.1371/journal.pone.0155457 27275601 PMC4898739

[B49] Trujillo P. Summers P. E. Ferrari E. Zucca F. A. Sturini M. Mainardi L. T. (2017). Contrast mechanisms associated with neuromelanin-MRI. *Magn. Reson. Med.* 78 1790–1800. 10.1002/mrm.26584 28019018

[B50] van der Pluijm M. Cassidy C. Zandstra M. Wallert E. de Bruin K. Booij J. (2021). Reliability and Reproducibility of Neuromelanin-Sensitive Imaging of the Substantia Nigra: A Comparison of Three Different Sequences. *JMRI* 53 712–721. 10.1002/jmri.27384 33037730 PMC7891576

[B51] Wang W. Zhornitsky S. Zhang S. Li C. R. (2021). Noradrenergic correlates of chronic cocaine craving: neuromelanin and functional brain imaging. *Neuropsychopharmacology* 46 851–859. 10.1038/s41386-020-00937-9 33408330 PMC8027452

[B52] Ward W. C. Zucca F. A. Bellei C. Zecca L. Simon J. D. (2009). Neuromelanins in various regions of human brain are associated with native and oxidized isoprenoid lipids. *Arch. Biochem. Biophys.* 484 94–99. 10.1016/j.abb.2009.01.013 19467634

[B53] Weinstein J. J. Chohan M. O. Slifstein M. Kegeles L. S. Moore H. Abi-Dargham A. (2017). Pathway-Specific Dopamine Abnormalities in Schizophrenia. *Biol. Psychiatry* 81 31–42. 10.1016/j.biopsych.2016.03.2104 27206569 PMC5177794

[B54] Wengler K. He X. Abi-Dargham A. Horga G. (2020). Reproducibility assessment of neuromelanin-sensitive magnetic resonance imaging protocols for region-of-interest and voxelwise analyses. *NeuroImage* 208:116457. 10.1016/j.neuroimage.2019.116457 31841683 PMC7118586

[B55] Zecca L. Bellei C. Costi P. Albertini A. Monzani E. Casella L. (2008). New melanic pigments in the human brain that accumulate in aging and block environmental toxic metals. *Proc. Natl. Acad. Sci. U.S.A.* 105 17567–17572. 10.1073/pnas.0808768105 18988735 PMC2582310

[B56] Zecca L. Fariello R. Riederer P. Sulzer D. Gatti A. Tampellini D. (2002). The absolute concentration of nigral neuromelanin, assayed by a new sensitive method, increases throughout the life and is dramatically decreased in Parkinson’s disease. *FEBS Lett.* 510 216–220. 10.1016/s0014-5793(01)03269-0 11801257

[B57] Zecca L. Stroppolo A. Gatti A. Tampellini D. Toscani M. Gallorini M. (2004). The role of iron and copper molecules in the neuronal vulnerability of locus coeruleus and substantia nigra during aging. *Proc. Natl. Acad. Sci. U.S.A.* 101 9843–9848. 10.1073/pnas.0403495101 15210960 PMC470762

[B58] Zecca L. Tampellini D. Gerlach M. Riederer P. Fariello R. G. Sulzer D. (2001). Substantia nigra neuromelanin: structure, synthesis, and molecular behaviour. *Mol. Pathol.* 54 414–418. 11724917 PMC1187132

[B59] Zhang Y. Loonam T. M. Noailles P. A. Angulo J. A. (2001). Comparison of cocaine- and methamphetamine-evoked dopamine and glutamate overflow in somatodendritic and terminal field regions of the rat brain during acute, chronic, and early withdrawal conditions. *Ann. N.Y. Acad. Sci.* 937 93–120. 10.1111/j.1749-6632.2001.tb03560.x 11458542

[B60] Zucca F. A. Basso E. Cupaioli F. A. Ferrari E. Sulzer D. Casella L. (2014). Neuromelanin of the human substantia nigra: an update. *Neurotox. Res.* 25 13–23. 10.1007/s12640-013-9435-y 24155156

[B61] Zucca F. A. Segura-Aguilar J. Ferrari E. Muñoz P. Paris I. Sulzer D. (2017). Interactions of iron, dopamine and neuromelanin pathways in brain aging and Parkinson’s disease. *Prog. Neurobiol.* 155 96–119. 10.1016/j.pneurobio.2015.09.012 26455458 PMC4826627

